# The CYP303A1 is essential in embryonic development of *Nilaparvata lugens* Stål (Hemiptera: delphacidae)

**DOI:** 10.3389/fphys.2025.1679768

**Published:** 2025-10-08

**Authors:** Yingzhen Liu, Zihan Yan, Di Deng, Huaiqi Wang, Ziyue Mao, Enrui Tang, Junyi Zhong, Xudong Zhao, Linquan Ge

**Affiliations:** ^1^ College of Plant Protection, Yangzhou University, Yangzhou, China; ^2^ Jiangsu Province Engineering Research Center of Green Pesticides, Yangzhou University, Yangzhou, China

**Keywords:** cytochrome P450, *CYP303A1*, *Nilaparvata lugens*, fecundity, embryonic development

## Abstract

The *N. lugens* is a highly fecund rice pest that causes severe damage to rice production in Asia. Cytochrome P450 monooxygenases (P450s) play essential roles in insect development, metabolism, and reproduction. In a previous study, we identified and functionally characterized CYP303A1, a member of the CYP2 family, in *N. lugens* molting and metamorphosis process. However, it is unclear the function of CYP303A1 in *N. lugens* fecundity. In this study, the expression profiling showed that *CYP303A1* is highly expressed in legs and adult fat bodies, with moderate expression in the ovaries. Silencing of *CYP303A1* in females had no significant effects on *Vg* (Vitellogenin) and *VgR* (Vitellogenin receptor) transcript levels, ovarian morphology, oocyte number, oviposition period, or female lifespan, indicating that CYP303A1 is not involved in vitellogenesis or ovarian development. However, knockdown of *CYP303A1* significantly prolonged the embryonic period and reduced egg hatchability. Morphological observations revealed that silencing *CYP303A1* led to abnormal embryonic development, including delayed eyespot formation and dispersed yolk granules. Furthermore, the expression levels of several egg hatchability-related genes, including *Cpr52*, *TwdIE3*, *HNF4*, and *Let1*, were significantly altered following *CYP303A1* silencing. These findings suggest that CYP303A1 is essential for successful embryogenesis in *N. lugens*, likely by regulating the expression of hatching-related genes. This work expands our understanding of the non-Halloween CYP genes in insect reproduction and provides a potential molecular target for disrupting *N. lugens* population development.

## 1 Introduction

Cytochrome genes encoding P450 enzymes are consistently found among the most prominent gene families in plants, animals and fungi (Dermauw et al., 2020; Nauen et al., 2022). Cytochrome P450 enzymes (CYP), commonly called CYP450s, play a crucial role in essential biological processes across a diverse range of organisms (Dermauw et al., 2020). In insects, these enzymes significantly impact physiological processes such as development, reproduction, detoxification, and resistance to pesticides (Nebert and Dalton, 2006; Feyereisen, 2012). Moreover, CYP450s are at the interface of environmental responses, metabolism, and endocrine regulation by catalyzing the transformation of a myriad of exogenous and endogenous substrates by hydroxylation, epoxidation, dealkylations and a great variety of other reactions (Ye et al., 2022; Tzotzos, 2025). In insects, CYP genes are mainly categorized into six clans: clan 2, clan 3, clan 4, and mitochondrial clan with two new additions clan 16 and clan 20, which are restricted to certain Apterygotes and Paleoptera with unknown functions (Dermauw et al., 2020). Most of the single-copy genes belong to the CYP2 clan and the mitochondrial CYP clan, and most of the multiple-copy paralogs belong to the CYP3 and CYP4 clans and are often arrayed in clusters on chromosomes (Feyereisen, 2011; 2012). Many closely paralogous genes are part of lineage-specific family expansions, called P450 blooms (Sezutsu et al., 2013; Hafeez et al., 2022). The P450s implicated in xenobiotic metabolism and pesticide resistance are often found in such blooms.

The CYP3 and CYP4 clans are generally predominant in insects and relate to insect chemical defence. Considerable evidence has shown that P450s of the CYP3 clan, represented by members of CYP6 and CYP9 families, are mainly involved in xenobiotic metabolism by direct detoxification (Sezutsu et al., 2013; Nauen et al., 2022). In addition, members of the CYP4 clan (in the CYP4G subfamily) participate in this process by adjusting cuticle penetration through biosynthesis of cuticular hydrocarbons (Balabanidou et al., 2016; Feyereisen, 2020). In contrast, the evolution and function of most P450s in CYP2 and mito clans are considered highly conserved, and they form many families with few or even single members. Definite evidence has linked P450s in these two clans to biosynthesis or metabolism of endogenous compounds in model insect species. The CYP2 in insects represents a highly conserved group of enzymes that play essential roles in multiple physiological processes. Functionally, CYP2 family members are primarily involved in the biosynthesis and metabolism of endogenous hormones, particularly ecdysteroids and juvenile hormones, which are critical for regulating insect molting, metamorphosis, reproduction, and development (Guittard et al., 2011; Truman, 2019; Jin et al., 2023; Wu et al., 2023). Several key CYP2 genes, such as CYP307A1 (Spook), CYP306A1 (Phantom), CYP302A1 (Disembodied), CYP315A1 (Shadow), and CYP314A1 (Shade), constitute the core of the so-called Halloween gene cluster and catalyze successive steps in the ecdysteroid biosynthetic pathway (Peng et al., 2019; Zhou et al., 2020; Yan et al., 2023). In addition to the previously defined Halloween genes, CYP18A1, which belongs to the CYP2 clan, is involved in 20E inactivation (Rewitz et al., 2010; Guittard et al., 2011). CYP301A1, which belongs to the mitochondrial CYP clan, was described as an important gene involved in the formation of the adult cuticle, but its biochemical function is unknown (Sztal et al., 2012). Another CYP2 clan member, CYP303A1 (nompH) is expressed in the socket cells of sensory bristles in *D. melanogaster*, and is essential for the development and structure of external sensory organs (Willingham and Keil, 2004). CYP303A1 is a strongly supported clade with generally a single gene for each species, but it is duplicated in the Argentine ant, *Linepithema humile*, and in the carpenter ant *Camponotus floridanus* where the two genes are in a tandem array. It is also duplicated in the damselfly *C. splendens*. CYP303A1 was not found in genomes or transcriptome shotgun assembly (TSA) beyond winged insects, and CYP303A1 is mostly a single copy gene, “stable” in insects (Fallon et al., 2018; Dermauw et al., 2020).

The brown planthopper, *N. lugens* Stål (Hemiptera: Delpahacide) is one of the most destructive insect pests in Asian rice-growing regions (Wu J. et al., 2020; Sun et al., 2024). It damages rice through direct phloem feeding and transmits Southern rice grassy stunt virus (RGSV), and rice ragged stunt virus (RRSV), leading to plant stunting, reduced tillering, and severe yield losses (Zhang et al., 2022; Zhu et al., 2023). In recent decades, the extensive use of chemical insecticides has not only disrupted natural enemy populations but also accelerated the evolution of insecticide resistance in *N*. *lugens* (Wei et al., 2009; Gao et al., 2025). Furthermore, its high reproductive capacity, strong migratory behavior, and adaptability to different rice cropping systems have made its management increasingly challenging (Wu J. et al., 2020; Zhao et al., 2025a). These factors highlight the urgent need to develop sustainable and ecologically sound control strategies against this pest. In our previous study, we found that CYP303A1 was crucial in *N. lugens* molting and metamorphosis process. Silencing of *CYP303A1* disrupted the synthesis of 20E in nymphs, caused downregulation of the 20E signaling pathway, and further affected the transcription of cuticular proteins and chitin metabolism, which ultimately affected the shedding of the old epidermis and the formation of the new epidermis (Wu et al., 2024). Ecdysteroids are considered as the classic insect hormones involved in oogenesis of insects (Song and Zhou, 2020; Benrabaa et al., 2022; Schellens et al., 2022). However, it is unclear whether CYP303A1 affects the embryonic development of *N. lugens* female adults. In this study, we systematically investigated the key role of CYP303A1 in the reproduction of *N. lugens* female adults, that it could serve as a potential RNAi target for green control of *N. lugens*, and making it an important target for both fundamental research and the development of novel pest control strategies.

## 2 Materials and methods

### 2.1 Insect culture

The *N. lugens* populations were originally obtained from the China National Rice Research Institute (CNRRI, Hangzhou, China). The strains were routinely reared on rice seedlings (Wuyunjing 23) under temperature (26 °C ± 2 °C), relative humidity (80% ± 10%), and photoperiod (light: dark, 16:8 h) as previously described by Wu et al. (2024).

### 2.2 The tissue-specific expression analysis of CYP303A1 in *N. lugens* females

The tissue samples (including the head, leg, cuticle, fatbody, and ovary) were dissected from virgin female adults at 2 days after emergence under a binocular microscope (Leica EZ4, Germany) by sterilized scalpel and tweezers on ice. Each sample was collected in triplicate from 30 females. The total RNA of each tissue sample was isolated using the RNA Easy Fast Tissue/Cell Kit (Tiangen, Beijing, China) according to the manufacturer’s instructions. The First-strand cDNA was synthesized using the PrimeScriptTM 1st Strand cDNA Synthesis Kit (TaKaRa, Dalian, China) according to the manufacturer’s instructions. The real-time quantitative PCR (RT-qPCR) was performed in CFX96 Touch Real-Time PCR Detection System (Bio-Rad Co., Ltd., CA, United States) in 10 μL reaction mixtures containing 5 μL 2× SYBR Premix EX TaqII Master Mix (TaKaRa, Dalian, China), forward and reverse primers of 0.2 μL (10 nM), 1 μL cDNA template and 3.6 μL ddH_2_O. The RT-qPCR of thermal cycling conditions were as follows: 95 °C for 4 min, 35 cycles of 95 °C for 10 s, 58 °C for 30 s, 72 °C for 20 s, with a final extension of 72 °C for 10 min. Subsequently, a melting curve analysis was conducted in the 60–95 °C temperature range to verify the consistency and specificity of each reaction product. RT-qPCR primers of *CYP303A1* were designed online (https://www.primer3plus.com/) and listed in Supplementary Table S1. The β-actin was used as a reference gene (Ge et al., 2020), and the relative gene expression was calculated using the 2^−ΔΔCT^ method (Livak and Schmittgen, 2001).

### 2.3 The dsRNA synthesis and microinjection

The double-stranded RNA (dsRNA) was synthesized by amplifying a CYP303A1 with a 333 bp fragment by T7 RNA polymerase promoter-linked primers, according to Wu et al. (2024). The Green Fluorescent Protein (GFP) was a negative control. All dsRNAs were synthesized using a T7 RiboMAX™ Express RNAi System (Promega, Madison, WI). Templates for the dsRNA synthesis were reacted in a thermal cycler (BIO-RAD, Hercules, CA, United States) following the procedure: 35 cycles at 95 °C for 30 s, 60 °C for 30 s, and 72 °C for 45 s, with a final extension at 72 °C for 10 min. PCR products were used as templates for dsRNA synthesis through purification kits (Novozymes Biotechnology, Nanjing, China), and the synthesized product was stored at −80 °C until use.

After dsRNA synthesis, the newly-emerged *N. lugens* females were anesthetized with CO_2_ and then injected with 100 ng, 150 ng, and 200 ng of dsCYP303A1 or dsGFP in the mid-thorax of female adults using a Naoject II microinjection device (Drummond Scientific, PA, United States) under a microscope. Then, the surviving females were transferred into a plastic cup (15 × 18 cm) containing rice plants at a 4-leaf stage (Zhu et al., 2023). Previous studies have demonstrated that *N. lugens* is susceptible to RNAi, and the silencing effect of dsRNA to target genes remained stable and highly efficient at 48 h, 72 h, 96 h, and even up to 120 h post-injection (Ge et al., 2015; Li et al., 2025). Therefore, the female adults were collected at 48 h post-injection of dsRNAs as a representative time point to examine the efficiency of RNAi. Each treatment contained at least 5 individuals and three independent biological replicates.

### 2.4 The isolation and observation of female adult ovaries

The ovaries of *N. lugens* dsRNAs-injected females were isolated and observed at 2 days (2 DAE) and 4 days (4 DAE) after emergence. The female adults were anesthetized with CO_2_ and subsequently dissected in 0.9% saline solution, and the fat bodies around the ovary were stripped cleanly. The isolated ovaries were washed and photographed using a microscope with a digital camera (Olympus, model SZX23, Japan). The count of ovarioles was documented using microscopic examination. There were 15 females used for each treatment.

### 2.5 Determine the reproduction and population parameters of *N. lugens*

The *N. lugens* reproductive parameters, including pre-oviposition periods, oviposition periods, and the number of eggs laid, were determined, referring to Ge et al. (2020), with slight variations. After 200 ng dsRNAs injected into newly newly-emerged females, the surviving females were paired 1:2 with untreated males (dsCYP303A1♀ × Control♂ or dsGFP♀ × Control♂) and were reared in glass tubes (2.5 cm diameter, 15 cm height) containing tillering rice stems. After the offspring (F1 generation) of *N. lugens* had reached the 3^rd^ instar, the number of offspring was counted and transferred to new rice stems, which were placed in glass cups and fed until emergence. After that, the unhatched eggs laid by the F0 generation on the rice stems were recorded. The hatching rate was calculated as offspring/offspring + unhatched eggs. The population growth index (PGI) was calculated as F_1_/F_0_, with F_1_ representing the total offspring of the following generation and F_0_ representing the number of parents (F0 = 4) (Zhao et al., 2025b). The rice stems were replaced every 24 h during the pre-oviposition period and every 48 h during the oviposition period until the females died. The pre- and oviposition periods and the number of eggs laid by females were recorded, and 15 replicates were used for each treatment.

### 2.6 The observation of eggs females laid, and assessment of expression levels of egg hatchability-related genes in *N. lugens* female adults

After the anesthetizing effect of dsRNAs-injected females vanished, the survived females were transferred into glass tubes (2.5 cm diameter, 15 cm height) containing 2–3 tillering rice stems and paired with untreated males at 1:2 ratio (female to male). The rice stems were replaced every 24 h during the pre-oviposition periods. Embryonic development of eggs can be accurately assessed using morphological landmarks. Previous studies have demonstrated that eyespot formation and yolk distribution patterns are reliable indicators of embryonic developmental progress and can be used to evaluate developmental status or delays in insect eggs (Panfilio, 2008; Fan et al., 2020). In this study, after the females laid eggs, rice stems containing eggs laid by the *N. lugens* females were collected at 2, 5, and 8 days after oviposition. The eggs laid by females in the rice stems were dissected, and the morphological characteristics of the egg development were observed through a microscope (OLYMPUS CX23 Japan), and photographed and recorded with a DS-Fi2 digital camera (Nikon Tokyo Japan). The newly emerged females were injected with dsRNAs as described in the previous sections, and the surviving female adults were collected at 2 days and 4 days post-emergence qRT-PCR and the procedure as mentioned above were used to determine the expression levels of egg hatchability-related genes (*Cpr3*, *Cpr8*, *Cpr10*, *Cpr24*, *Cpr36*, *Cpr47*, *Cpr51*, *Cpr52*, *Cpr54*, *Cpr58*, *Cpr73*, *Cpr90*, *Cpr94*, *TwdIE3*, *CPAP1-E*, *CPAP1-H*, *CPAP1-I*, *CPAP3-B*, *CPAP3-D1*, *HNF4*, *Hox3*, and *Let1*) (Pan et al., 2018; Ren et al., 2018; Cheng et al., 2020; Lu et al., 2023). The treatment and control consisted of three independent biological replicates, and each replicate was composed of 10 individuals.

### 2.7 Data analysis

Student's t-tests were used to compare statistical differences in the gene expression levels and biological parameters of *N. lugens* between the control and gene-silenced groups. One-way analysis of variance (ANOVA) by the Tukey test was used to compare statistical differences in the gene expression levels of *CYP303A1* in different tissues. All statistical tests were conducted in SPSS 22.0 (IBM Inc., Armonk, NY, United States), and plots were generated using Origin 2023 (OriginLab Inc., Northampton, United Kingdom).

## 3 Results

### 3.1 The tissue-specific expression profile and RNAi efficiency of CYP303A1 in *N. lugens* females

**FIGURE 1:**
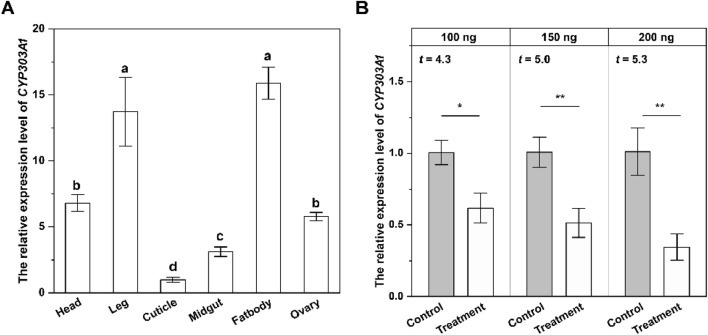
The tissue-specific expression level **(A)** and silencing efficiency of *CYP303A1* **(B)** in females. All data are means ± SE. The different lowercase letters represent statistically significant differences in the gene expression levels of *CYP303A1* in different tissues by the Tukey test. The asterisks (*P < 0.05, **P < 0.01) represent statistically significant differences between control and treatment by Student’s t-test.

To investigate the physiological function of CYP303A1 in *N. lugens* females, we analysed the expression profiles of CYP303A1 in female different tissues by RT-qPCR. The results showed that the expression level of *CYP303A1* was significantly different in various tissues (*F* = 52.2, *P* < 0.001), and was predominantly enriched in female fatbodies (Fb) and leg, and showed a lower transcript level in cuticle (Figure 1A).

We further determined the RNAi efficiency of CYP303A1 in females. Compared with the dsGFP-injected females, the gene expression levels of newly emerged females injected with 100 ng, 150 ng and 200 ng of dsCYP303A1 decreased by 38.8% (*t* = 4.3, *P* < 0.05), 49.3% (*t* = 5.0, *P* < 0.01) and 66.6% (*t* = 5.3, *P* < 0.01), respectively, at 2 days. Based on the silencing efficiency, 200 ng of dsCYP303A1 was selected for subsequent experiments (Figure 1B).

### 3.2 Effect of silencing *CYP303A1* on ovarian development in female adults

**FIGURE 2:**
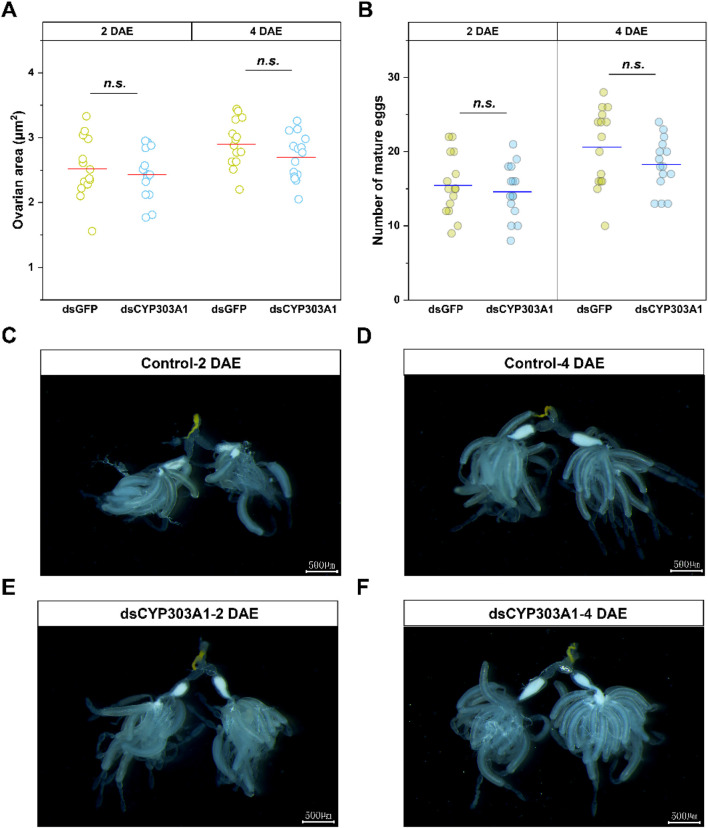
Effects of dsCYP303A1 on ovarian area **(A)**, number of mature eggs **(B)**, and ovary development **(C–F)** of *N. lugens* females. The ns represent no significant differences in expression level between control (dsGFP) and treatment (dsCYP303A1) by Student's t-test.

We further analyzed the effect of silencing *CYP303A1* on the ovarian development of *N. lugens* females. The results showed that the mean ovarian area (Figures 2A DAE: *t* = 0.6, *P* > 0.05; 4 DAE: *t* = 1.4, *P* > 0.05) as well as the number of mature eggs (Figures 2B DAE: *t* = 0.4, *P* > 0.05; 4 DAE: *t* = 1.0, *P* > 0.05) were reduced in CYP303A1-suppressed females than dsGFP-injected females at 2, and 4 days post-emergence, but there were no statistical differences. Moreover, the morphological observations showed that the ovaries of both dsGFP-injected and dsCYP303A1-injected females were completely filled with regular banana-shaped oocytes, which were closely arranged in the ovarioles at 2 and 4 DAE (Figures 2C–F). These results indicated that suppression of *CYP303A1* might be involved in the cuticle barrier construction. These results indicated that suppression of *CYP303A1* would not disrupt ovarian development in *N. lugens* females.

### 3.3 Silencing of *CYP303A1* decreased the number of offspring in *N. lugens*

Subsequently, we statistically examined the reproductive and population parameters of *N. lugens* after CYP303A1 silencing in females. The results showed that reproductive parameters, including number of eggs laid (Table 1, *t* = 0.34, *P* > 0.05), pre-oviposition (*t* = 0.27, *P* > 0.05), oviposition periods (*t* = 1.47, *P* > 0.05), and female longevity (*t* = 0.44, *P* > 0.05) were not significantly different between dsCYP303A1-injected *N. lugens* and controls (Table 1). However, silencing *CYP303A1* significantly affected the population parameters of *N. lugens*. Inhibition of *CYP303A1* significantly prolonged the egg durations by 47.3% and significantly reduced egg hatchability by 68.6% compared to the controls (Table 1). Ultimately, the population parameters of dsCYP303A-injected *N. lugens* were significantly reduced by 68.1% compared to the control (Table 1). These results demonstrate that inhibition of *CYP303A1* will not affect female ovary development, but will reduce the number of offspring by decreasing egg hatchability of *N. lugens*.

**TABLE 1:** Effects of silencing CYP303A1 on the reproductive and population parameters of *Nilaparvata lugens*.

Reproductive and population parameters	dsGFP	dsCYP303A1
Number of eggs laid	241.2 ± 41.9	246.8 ± 47.4
Preovipositon periods	4.0 ± 0.7	4.1 ± 0.7
Ovipositon periods	15.0 ± 1.3	14.3 ± 1.3
Female longevity	18.7 ± 1.2	18.5 ± 1.2
Egg durations	9.7 ± 0.8 b	14.3 ± 1.5 a
Hatching rate (%)	83.3% ± 2.0% a	26.2% ± 10.3% b
Sex ratio (female to male)	1.2 ± 0.1	1.2 ± 0.1
Population growth index (PGI)	100.5 ± 17.8 a	32.0 ± 3.6 b

All data are means ± SE., The different lowercase letters represent statistically significant differences between control and treatment by Student’s t-test.

### 3.4 Silencing of *CYP303A1* delayed the egg development of *N. lugens*

**FIGURE 3:**
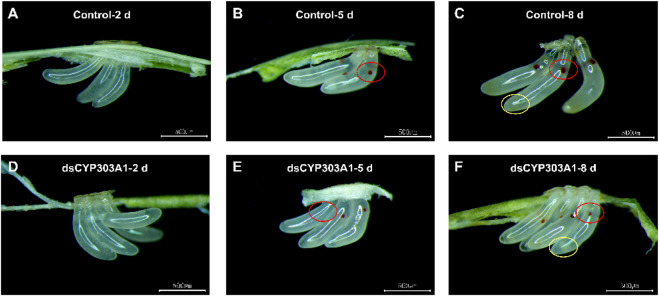
Effects of silencing *CYP303A1* on embryonic development in *N. lugens*. Note: **(A)**: Eggs laid for 2 days by dsGFP-treated females; **(B)**: Eggs laid for 5 days by dsGFP-treated females; **(C)**: Eggs laid for 2 days by dsGFP-treated females; **(D)**: Eggs laid for 2 days by dsCYP303A1-treated females; **(E)**: Eggs laid for 5 days by dsCYP303A1-treated females; **(F)**: Eggs laid for 8 days by dsCYP303A1-treated females. Red circle in the figure mark embryo eyespots, yellow circle in the figure mark aggregated substance in the eggs.

The silencing of *CYP303A1* significantly delayed the egg development of *N. lugens*. The eggs laid by dsCYP303A1- and dsGFP-treated females showed no obvious phenotypic differences at 2 days after oviposion (Figures 3A,D). Compared with the dsGFP-treated females, some eggs did not develop eyespots after 5 days of egg laying in dsCYP303A1-treated newly emerged females (Figures 3B,E). In dsGFP-treated newly emerged females, embryonic eye spots developed normally after 8 days of egg laying, and a distinct yellow substance was produced and accumulated at the egg tip (Figure 3C). In contrast, in dsCYP303A1-treated newly emerged females, embryonic eye spot development was delayed after 8 days of egg laying, and the egg tip exhibited a transparent substance (Figure 3F).

### 3.5 Suppression of *CYP303A1* reduced the transcript level of hatchability-related genes in *N. lugens* female adults

**FIGURE 4:**
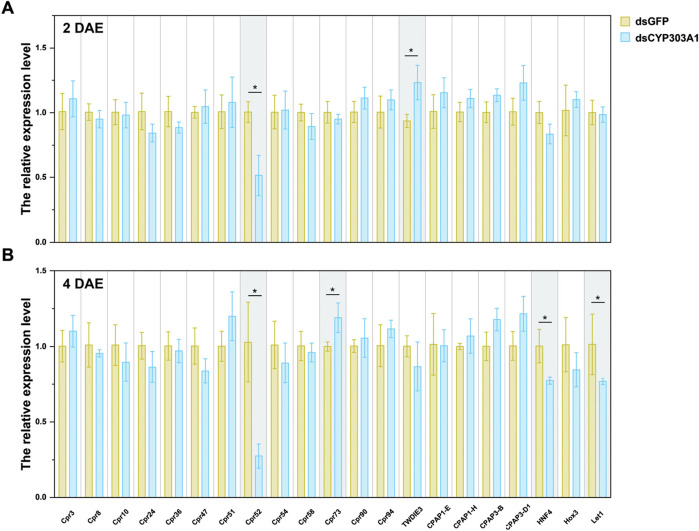
Effects of silencing CYP303A1 on the expression levels of genes involved in embryonic development in *N. lugens*. Note: **(A)** The expression levels of hatchability-related genes in females at 2 days post-eclosion. **(B)** The expression levels of hatchability-related genes in females at 4 days post-eclosion. All data are means ± SE. The asterisks (*P < 0.05) represent statistically significant differences between control and treatment by Student’s t-test.

Finally, we investigated the effects of *CYP303A1* knockdown on the expression of egg-hatching-related genes in female *N. lugens*. These genes included *Cpr3*, *Cpr8*, *Cpr10*, *Cpr24*, *Cpr36*, *Cpr47*, *Cpr51*, *Cpr52*, *Cpr54*, *Cpr58*, *Cpr73*, *Cpr90*, *Cpr94*, *TwdIE3*, *CPAP1-E*, *CPAP1-H*, *CPAP1-I*, *CPAP3-B*, *CPAP3-D1*, as well as transcription factors *HNF4*, *Hox3*, and *Let1.* The results showed that injection of dsCYP303A1 significantly downregulated the expression level of *Cpr52* by 49.0% (Figure 4A, *t* = 4.2, *P* < 0.05) and upregulated *TwdIE3* expression by 32.0% (Figure 4A, *t* = 3.1, *P* < 0.05) in females at 2 days post-eclosion. At 4 days post-eclosion, the expression levels of *Cpr52*, *HNF4*, and *Let1* were reduced by 75.2% (Figure 4B, *t* = 4.1, *P* < 0.05), 22.8% (Figure 4B, *t* = 3.03, *P* < 0.05), and 32.0% (Figure 4B, *t* = 2.87, *P* < 0.05), respectively, in dsCYP303A1-treated females compared to the dsGFP controls. These findings suggest that silencing *CYP303A1* markedly suppresses the expression of genes associated with egg hatching, thereby impairing embryonic development and hatching rate.

## 4 Discussion

In oviparous insects, reproduction is a complex process involving multiple sequential stages, including previtellogenesis, vitellogenesis, chorion formation, oocyte maturation, and egg hatching (Cheng et al., 2020). To date, most cytochrome P450 (CYP) genes known to regulate insect reproduction are members of the Halloween gene family, which are essential for the biosynthesis of 20E (Lao et al., 2015; Truman, 2019; Hafeez et al., 2022). Functional studies have shown that silencing CYP307A2, CYP302A1, or CYP314A1 in *N. lugens* results in defective ovarian and egg development and markedly reduced fecundity (Zhou et al., 2020). Similarly, mutations in CYP306A1 or CYP302A1 in *Drosophila melanogaster* cause embryonic lethality (Chávez et al., 2000; Niwa and Niwa, 2016). In *Schistocerca gregaria*, RNAi knockdown of *CYP307A1*, *CYP306A1*, or *CYP314A1* disrupts oocyte development and leads to reduced egg production and hatching success (Schellens et al., 2022). Ecdysteroids are aslso considered as the classic insect hormones involved in oogenesis of insects; however, their function and importance differ between distinct insect orders (Song and Zhou, 2020; Khalid et al., 2021). Ecdysteroids are needed to establish and maintain the stem cell niche (Gancz et al., 2011), to stimulate follicle cell formation and border cell migration (Morris and Spradling, 2012), to coordinate the onset of vitellogenesis with the availability of nutrients, to stimulate yolk polypeptide synthesis in the fat body, and finally, to induce choriogenesis (Buszczak et al., 1999; Carney and Bender, 2000; Terashima and Bownes, 2004). In previous study, we demonstrated that CYP303A1 plays a critical role in molting and metamorphosis of *N. lugens*. CYP303A1 was highly expressed during the pre-molt stage and primarily localized in tissues associated with cuticle formation. RNAi-mediated silencing of *CYP303A1* significantly reduced the titer of 20E and downregulated key genes in the 20E signaling pathway, leading to impaired transcription of cuticular proteins and disrupted molting and metamorphic processes. These results indicate that CYP303A1 exerts a central role in molting and metamorphosis by regulating 20E signaling. Notably, the functions of CYP303A1 in other physiological processes of *N. lugens* have not been reported. Given the critical role of 20E in female reproduction and ovary development, CYP303A1 is likely to influence reproductive capacity and ovarian development through modulation of 20E biosynthesis.

*N. lugens* is a highly fecund r-strategy insect, represents a persistent threat to rice production and agroecosystem sustainability (Hu et al., 2017; Gou et al., 2024). To date, 67 CYP450 genes have been identified in *N. lugens* (Xue et al., 2014). Among these, conserved CYP genes from the CYP2 family and mitochondrial CYP clan have been implicated in reproductive regulation (Li et al., 2015; Zhou et al., 2020). Previous studies have demonstrated that Halloween genes play key roles in regulating Vg and VgR expression, oocyte development, and embryogenesis (Peng et al., 2019; Wu Z. et al., 2020; Benrabaa et al., 2022; Jin et al., 2023). Intriguingly, recent findings suggest that CYP genes outside the Halloween family may also contribute to insect reproduction. For instance, CYP303A1 has been implicated in embryonic development in *D. melanogaster*, where its mutation leads to impaired dorsal vessel formation and late-stage embryonic arrest, resulting in lethality (Wu et al., 2019). However, whether CYP303A1 is involved in embryogenesis and reproductive regulation in hemipteran pests such as *N. lugens* remains unclear. In the current study, CYP303A1 is abundantly expressed in the fat body of adult females and exhibits moderate expression in the ovary, suggesting a potential role in ovarian or embryonic development. Interestingly, although CYP303A1 was highly expressed in the fat body, RNAi knockdown of *CYP303A1* did not significantly affect the transcript levels of *Vg* or *VgR*, nor did it impact ovarian morphology, the number of mature oocytes, ovarian size, oviposition parameters, female longevity, or progeny sex ratio. This discrepancy suggests that CYP303A1 may execute tissue-specific roles distinct from yolk protein synthesis. In the fat body, CYP303A1 might participate in processes such as ecdysteroid biosynthesis, lipid mobilization, or nutrient homeostasis, thereby indirectly contributing to reproductive fitness. By contrast, in embryos, its role appears to be directly associated with embryogenesis and successful hatchability through the regulation of developmental gene networks, and previous studies in *N. lugens* have shown that CYP303A1 is highly expressed in eggs (Wu et al., 2024). In *D. melanogaster*, CYP303A1 was predominantly enriched in the ring gland, and was essential in embryonic development (Wu et al., 2019). It also indicated the functional diversification of P450 genes.

Moreover, we further found that silencing *CYP303A1* significantly prolonged the egg developmental period and markedly reduced egg hatchability. The embryonic development of *N. lugens* eggs exhibits clear stage-specific characteristics and is closely temperature-dependent (Fan et al., 2020). At room temperature, the egg period lasts ∼192 h from oviposition to first-instar nymph hatching. In early embryogenesis (0–6 h after egg laying, AEL), eggs appear oyster white and gradually turn yellow, with synchronous nuclear divisions forming a syncytial stage, providing an optimal window for RNAi or genome-editing (Huang et al., 2016). By 30 h AEL, the germ band forms, the embryo elongates and segments (intermediate germ development). Eyespots appear at 96 h AEL, darken at 120 h, abdominal segments form sequentially, appendages complete by 168 h, and nymphs hatch at 192 h AEL (Fan et al., 2020). Egg length and width increase throughout development, reflecting rapid cellular proliferation and morphological remodeling. In the current study, the eggs from dsCYP303A1-treated females exhibited delayed eyespot development and transparent terminal yolk structures that failed to aggregate and appear yellow, strongly suggesting that CYP303A1 plays a critical role in embryonic development in *N. lugens*. In *Locusta migratoria, CYP303A1* is mainly expressed at fourth- and fifth-day of the egg stage (Zhang et al., 2018). Interestingly, although CYP303A1 is conserved across insects, its knockdown produces divergent phenotypes between different species. In *N. lugens*, eggs are laid externally on plant tissues, and early embryogenesis requires the formation of a serosal cuticle to protect the embryo and support nutrient allocation (Fan et al., 2020). Consequently, CYP303A1 may primarily influence embryonic morphogenesis. In contrast, in *D. melanogaster*, the developmental arrest occurred in the late embryonic development in the CYP303A1 mutants, showing an abnormal dorsal vessel (Wu et al., 2019). In holometabolous insects such as *Drosophila*, embryogenesis occurs within a more protected eggshell environment (Donoughe, 2022), and CYP303A1 may play a greater role in dorsal vessel development or hormonal regulation. These observations suggest a life-history–dependent functional diversification of CYP303A1. Compared to the known ecdysteroidogenic genes exhibiting a typical Halloween-class embryonic phenotype, the CYP303A1 mutants have a later stage of arrest in embryonic development after near completion of dorsal closure (Wu et al., 2019). In a previous study, we demonstrated that the knockdown of *CYP303A1* significantly downregulated thetranscript levels of ecdysteroid biosynthesis-related genes, CYP307A1 and CYP314A1, as well as reducing the 20E titers in *N. lugens* (Wu et al., 2024; Du et al., 2025). 20E is a key insect steroid hormone that plays vital roles in female reproduction, particularly in regulating oogenesis and embryogenesis. During oogenesis, 20E promotes vitellogenin synthesis in the fat body and facilitates its uptake into oocytes via vitellogenin receptors, thereby supporting yolk accumulation and oocyte maturation. In embryogenesis, maternally deposited 20E participates in coordinating crucial developmental events such as germband extension and cuticle formation (Swevers, 2019; Song and Zhou, 2020; Khalid et al., 2021; Schellens et al., 2022). In *S. gregaria*, depleting the expression of *SchgrSpo (CYP307A1)*, *SchgrSad (CYP315A1)* and *SchgrShd (CYP314A1)* had a significant impact on oocyte development, oviposition and hatching of the eggs. Moreover, the shape of the growing oocytes, as well as the deposited eggs, was very drastically altered by the experimental treatments (Schellens et al., 2022). In *Diaphorina citri*, inhibition of Halloween gene expression in adults impeded the growth of the female ovary, diminished yolk formation, lowered vitellogenin transcription levels, and impaired female fecundity (Zhang et al., 2023). In *Bombyx mori*, knockdown of a dephosphorylation enzyme of 20E delayed development at early embryogenesis, whereas knockdown of an ecdysteroidogenic enzyme delayed development at early-middle embryogenesis (Fujinaga et al., 2020). These results demonstrated the essential role of 20E in insect embryonic development and further suggest that *CYP303A1* might disturb the embryonic development of *N*. *lugens* by modulating the 20E signaling pathway.

Furthermore, we found that the expression of several genes associated with eggshell formation and embryonic development (*Cpr52*, *TwdIE3*, *HNF4*, and *Let1*) was significantly altered following *CYP303A1* knockdown. These findings suggest that CYP303A1 plays a crucial role in coordinating late-stage embryogenesis, likely by regulating hatching-related gene expression. Cuticular proteins (CPRs) are the major structural components of the insect cuticle and play indispensable roles in cuticle formation, mechanical support, and protection (Pan et al., 2018; Mallick and Eleftherianos, 2024). During embryogenesis, the expression of specific CPR genes is tightly regulated both spatially and temporally to coordinate the formation of embryonic cuticular structures such as the serosa cuticle, embryonic epidermis, and chorion (Pan et al., 2018). In the late stages of embryogenesis, CPRs contribute to key morphogenetic events, including dorsal closure, head involution, and the formation of body segmentation (Charles, 2010). Disruption of cuticular protein genes can impair these processes, leading to defects in embryo elongation, desiccation resistance, and ultimately hatching failure (Moussian et al., 2006). Many holo- and hemimetabolous insects enhance their eggshells during embryogenesis by forming a serosal cuticle. Previous work in *N*. *lugens,* five cuticle protein coding genes, Cpr1/2/3/8/90, were specifically or highly expressed during the serosal cuticle formation period. TEM observations of the SC following parental RNAi against NlugCpr1/2/3/8/90 demonstrated that NlugCpr3/8/90 were essential for serosal cuticle formation (Lu et al., 2022). The transcription factors, HNF4, and Let1 have been reported that were essential for embryonic development (Ren et al., 2018; Cheng et al., 2020). In *N*. *lugens*, *HNF4* was highly expressed in the fat body and ovary of females, and knockdown of *HNF4* resulted in a dramatic reduction in egg hatching rate (Cheng et al., 2020). The *Let1* accumulates during the serosal cuticle formation period in *N*. *lugens*, and is located in the serosal endocuticle. RNAi-mediated silencing of *Let1* disrupted the serosal cuticle structure, accompanied by a loss of the outward barrier and 100% embryo mortality (Lu et al., 2023). These results suggest that silencing *CYP303A1* altered the expression of several genes related to chorion formation and embryonic morphogenesis, further affecting embryonic development.

In conclusion, this study provides new insights into the role of CYP303A1, a non-Halloween cytochrome P450 gene, in the embryonic development of the *N. lugens,* and expands our understanding of P450 gene family diversity in insect reproductive biology beyond the well-studied Halloween genes. Although CYP303A1 is not involved in vitellogenesis or ovarian maturation, its high expression in eggs and fat body, along with RNAi-based functional analysis, indicates its essential role during embryogenesis. Knockdown of CYP303A1 significantly reduced hatchability and delayed embryonic development, accompanied by abnormal eyespot formation and yolk distribution. These findings indicate that CYP303A1 is indispensable for proper embryogenesis, likely through the regulation of hatching-related genes, and highlight its potential as a molecular target for RNAi-based pest control. However, the translation of such molecular targets into field-deployable RNA pesticides is constrained by delivery challenges. Naked dsRNA is inherently unstable under field conditions, rapidly degrading by UV radiation, rainfall, and nucleases, which greatly limits its persistence. Moreover, phloem-feeding insects such as planthoppers exhibit low uptake efficiency of exogenous RNA, making it difficult to reproduce laboratory efficacy in the field. Despite these hurdles, significant opportunities are emerging: spray-induced gene silencing (SIGS) provides a practical, non-transgenic approach for large-scale deployment, while nanocarrier-based systems can markedly improve dsRNA stability, cellular uptake, and trans-barrier transport (Hoang et al., 2022; Qiao et al., 2024). Integrating these delivery innovations with key developmental targets such as CYP303A1 may enable the transition of RNAi from laboratory proof-of-concept to effective field application, offering a sustainable strategy for *N. lugens* management.

## Data Availability

The original contributions presented in the study are publicly available. This data can be found here: [https://www.ncbi.nlm.nih.gov/nuccore/FJ907954.1]. Additional data can be requested to LG at lqge@yzu.edu.cn.
