# Supplementary material for: The CYP303A1 is essential in embryonic development of Nilaparvata lugens Stål (Hemiptera: delphacidae)
Source: Front Physiol. 2025 Oct 8;16:1679768. doi: 10.3389/fphys.2025.1679768 (PMC12540425; doi:10.3389/fphys.2025.1679768)
Supplement: Supplementary file 1 [file DataSheet1.docx]

**Supplementary materials:**

**Table S1. List of the primers used in this study.**

|  | Primer name | Primer sequence（5’-3’） |
| --- | --- | --- |
| RT-qPCR | Q- *CYP303A1*-F | GCCACAAGAATTTCGCCCTG |
|  | Q- *CYP303A1*-R | CTGCAGGAGAGTTGCAGTGA |
|  | Q-*Vg*-F | CCCTAGTCAGTCCAGTGCC |
|  | Q-*Vg-*R | TGGCGAGAGGAACTGTCA |
|  | Q-*VgR*-F | AGGCAGCCACACAGATAACCGC |
|  | Q-*VgR-*R | AGCCGCTCGCTCCAGAACATT |
|  | Q-*Cpr3-*F | CAGTGAGGTGCAGGATGAG |
|  | Q-*Cpr3-*R | TACTCCACCAGGACGTTGTC |
|  | Q-*Cpr8-*F | TGCTCCAGCACCCACCTACT |
|  | Q-*Cpr8-*R | CGTTGTTGTCGCGGATGTCG |
|  | Q-*Cpr10-*F | TACCTGAAGAACCCCGGCCA |
|  | Q-*Cpr10-*R | CCTCGGCGTGGAATCCTGTC |
|  | Q-*Cpr24-*F | GGAATAGCGGCCGACGAACA |
|  | Q-*Cpr24-*R | GTCAGCGGCATAGACCACCC |
|  | Q-*Cpr36-*F | TGGACGGTCACTTACTCATTCTT |
|  | Q-*Cpr36-*R | CCGCTGCTGTAGTCCTCATT |
|  | Q-*Cpr47-*F | CCGGATCGTCTTACTCTTCAGG |
|  | Q-*Cpr47-*R | ACGAGTAACCTCCTTTCAGAGC |
|  | Q-*Cpr51-*F | GCTCAGTACCTATCCGCACC |
|  | Q-*Cpr51-*R | GGCCTGCTTCACCTCTGATT |
|  | Q-*Cpr52-*F | GCCACCACTACGAAGAAG |
|  | Q-*Cpr52-*R | CTCCTCCTGCTCCTTGAT |
|  | Q-*Cpr58-*F | GAGACATCCACGCACAGGAG |
|  | Q-*Cpr58-*R | GGCGGTGTACTCGACAATCC |
|  | Q-*Cpr73-*F | AGGTGGTGGAGGAGGTAGCG |
|  | Q-*Cpr73-*R | CCTCGTCGTGGTGTTCCTCG |
|  | Q-*Cpr90-*F | TGCAGCCAGACGGTTTCAGG |
|  | Q-*Cpr90-*R | GACACGAGGCTGTTGCCCTT |
|  | Q-*Cpr94-*F | CATCCAGGCCGTCCATGACC |
|  | Q-*Cpr94-*R | TAGCCGTGGTGGGTGACTGA |
|  | Q-*TwdIE3-*F | ACTTCATCCGCTACAAGAC |
|  | Q-*TwdIE3-*R | CGATTCCGCCTCCAATG |
|  | Q-*CPAP1-H-*F | CGGGCCATCTGCTCCTGAAG |
|  | Q-*CPAP1-H-*R | GCCAGACTCCGTGTGGGATG |
|  | Q-*CPAP1-E-*F | GCGTCAGGGTTGCTGGTCTT |
|  | Q-*CPAP1-E-*R | AGATGGGTGGGGCACTGGAT |
|  | Q-*CPAP3-B-*F | AGTGCGACCTGCCCTTCAAC |
|  | Q-*CPAP3-B-*R | TGTCGCATACGTTCGCCTCC |
|  | Q-*CPAP3-D1-*F | CCTTCTATTGGCCGCCTCGG |
|  | Q-*CPAP3-D1-*R | ACGGAACCCTGTCCGTCGTA |
|  | Q-*HNF4*-F | CCCATGCAATGTGAAGTGCC |
|  | Q-*HNF4*-R | CATGGGCACACATACGTTGG |
|  | Q-*Hox3*-F | AACTTCAGTGCCAGGTCGAG |
|  | Q-*Hox3*-R | TGGCAGGCAAACTCTGGAAA |
|  | Q-*Let1*-F | TTGGCTCTCCTTTTGCCACT |
|  | Q-*Let1*-R | GAGAAGTAGTAGCCGTGCCC |
| Synthesis of dsRNA | T7-*CYP303A1*-F | TAATACGACTCACTATAGGG  TACCAGGCCGTCAGGGAGAT |
|  | T7-*CYP303A1*-R | TAATACGACTCACTATAGGG  AGCCAGCATGGTCCACAGAG |
|  | T7-*GFP*-F | TAATACGACTCACTATAGGG  AAGGGCGAGGAGCTGTTCACCG |
|  | T7-*GFP*-R | TAATACGACTCACTATAGGG  CAGCAGGACCATGTGATCGCGC |
